# Using ChatGPT as an assessment tool for medical residents in Mexico: a descriptive experience

**DOI:** 10.3389/frai.2025.1662203

**Published:** 2025-09-15

**Authors:** Cristian N. Rivera-Rosas, J. R. Tadeo Calleja-López, Sandra J. Larios-Camacho, Sergio Trujillo-López

**Affiliations:** ^1^General Hospital Zone 89, Mexican Social Security Institute, Guadalajara, Mexico; ^2^General Hospital Zone 14, Mexican Social Security Institute, Hermosillo, Mexico; ^3^General Hospital Zone 14, Mexican Social Security Institute, Guadalajara, Mexico; ^4^Department of Medicine and Health Sciences, University of Sonora, Hermosillo, Mexico

**Keywords:** ChatGPT, artificial intelligence, medical education, resident physicians, multiple choice question exams

## Abstract

**Introduction:**

Artificial intelligence (AI) in medical education has progressed gradually, with numerous authors debating whether to prohibit, restrict, or adopt its use in academic contexts. Growing evidence exists regarding the capabilities and applications of AI in this field, particularly in supporting educational tasks such as student assessment. In this article we described our experience using ChatGPT to evaluate medical residents.

**Materials and methods:**

A descriptive cross-sectional study was conducted involving 35 medical residents from different specialty’s at a secondary-level hospital. Two different exams were generated using ChatGPT in topics of Rocky Mountain Spotted Fever (RMSF) and *Pertussis*. Additionally, an opinion survey—previously validated was administered to assess participants’ perceptions of ChatGPT ability to generate multiple-choice questions.

**Results:**

Overall average score for the *Pertussis* examination was 8.46, while the average for the RMSF examination was 8.29. All participants reported that the examination was well written and that the language used was coherent; 34 residents (97.14%) stated that the language was clear, concise, and easy to understand; 9 residents (25.71%) agreed that the language used was confusing; 33 residents (94.28%) rated the exams questions as difficult; 32 residents (91.42%) felt that they had adequately prepared for both examinations.

**Discussion:**

ChatGPT exhibits a promising faculty as a tool to support teaching activities in the training of medical specialists, mainly in reducing the human workload of healthcare personnel, and becoming integral to the next phase of medical education through AI-assisted content creation supervised by educators.

## 1 Introduction

Large language models (LLMs) such as ChatGPT continue to revolutionize human activities across various clinical and professional domains. The integration of artificial intelligence (AI) into medical education has progressed gradually, with numerous authors debating whether to prohibit, restrict, or adopt its use in academic contexts (Sánchez Mendiola, 2023). Nonetheless, there is growing evidence regarding the capabilities and applications of AI in this field, particularly in passing medical licensing examinations and supporting educational tasks such as student assessment, clinical scenario development, and the creation of formative feedback (Rivera-Rosas et al., 2024; Aster et al., 2024). These developments have sparked renewed reflection on the future of medical education.

Despite the increasing anecdotal recognition of AI’s plausible utility in the training of medical residents and the educational responsibilities of faculty members, there remains a scarcity of empirical evidence documenting medical educators’ experiences with these tools to address learning needs inherent to medical residence training. LLMs like ChatGPT represent potentially transformative tools that could support a new paradigm in medical education by alleviating the workload of both faculty and trainees, particularly in routine academic tasks such as exam generation and assessment.

The objective of this article is to describe our experience utilizing ChatGPT for the creation of multiple-choice question (MCQ) exams administered to medical residents from various specialties at a secondary-level hospital in Mexico, as well as to report resident perceptions regarding the AI-generated questions.

## 2 Materials and methods

A descriptive cross-sectional study was conducted involving 35 medical residents from the specialties of anesthesiology, emergency medicine, and internal medicine at a secondary-level hospital in Mexico. Initially, a general session on the topic *Pertussis-like Syndrome and Pertussis* was delivered over the course of one week. One week later, a session was held on *Rocky Mountain Spotted Fever caused by Rickettsia rickettsii (RMSF)*.

To generate the exams, ChatGPT-3.5, in its “Scholar GPT” mode, was prompted using two different inputs (Table 1) to create two questionnaires consisting of 15 multiple-choice questions each, based on the material presented in the corresponding class (Supplementary Table 1). Subsequently, using the Delphi method, three physicians reviewed the AI-generated questions and selected 10 questions from each questionnaire. Items were excluded due to inappropriate focus, inaccuracies in the AI-generated answers, or misalignment with the content covered during the instructional sessions.

**Table 1:** Prompts used for generating MCQ exams.

Prompts used for generating MCQ exams	
Whooping Cough Exam	You are a physician and professor at a hospital. You are in charge of the teaching area and conducted a general session at the hospital where you work, training staff on the topic of Pertussis-like Syndrome and Whooping Cough. Create 15 multiple-choice questions covering the etiology, clinical presentation, diagnosis, and treatment of this topic. Each question should have 4 options and only one correct answer. Show me the correct answer.
RMSF Exam	You are a physician and professor at a hospital. You are in charge of the teaching area and conducted a general session at the hospital where you work, training staff on the topic of Rickettsiosis and Rocky Mountain Spotted Fever (*Rickettsia rickettsii*). Create 15 multiple-choice questions covering the etiology, clinical presentation, diagnosis, and treatment of this topic. Each question should have 4 options and only one correct answer. Show me the correct answer.

RMSF, Rocky Mountain Spotted Fever; MCQ, multiple-choice questions.

Additionally, an opinion survey—previously validated in a Mexican student population was administered to assess participants’ perceptions of ChatGPT-3.5’s ability to generate multiple-choice questions (Rivera-Rosas et al., 2024). The survey responses were later dichotomized for analysis. Importantly, none of the participants were aware that the exam questions had been generated using ChatGPT-3.5.

**Figure 1:**
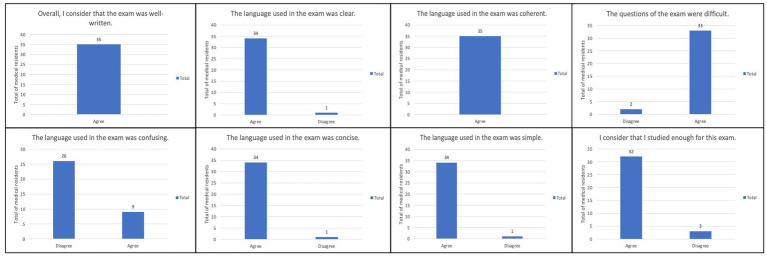
Overall results from the satisfaction survey used for medical residents’ perception about the exams’ questions. *n* = 35.

After the final selection of questions for each exam and one week after the RMSF session, the two separate exams created with ChatGPT-3.5 were administered to the residents covering the respective topics previously mentioned. The Google Forms platform was used to administer both the assessments and the opinion survey to the residents. When answering the google forms survey, residents were first asked to give their consent for using the results of their responses for academic and research purposes. The evaluation results were recorded in a Microsoft Office Excel 360® spreadsheet. Descriptive statistical analysis was conducted using frequency measures, and the results were presented through bar charts (Figure 1). No additional statistical tests were performed.

## 3 Results

A total of two assessments were administered to 35 medical residents at various stages of specialty training. Of these, 18 were male (51.43%) and 17 were female (48.57%). Regarding specialty distribution, 8 residents were from anesthesiology (22.86%), 20 from emergency medicine (57.14%), and 7 from internal medicine (20.0%). On a scale of 1 to 10, the overall average score for the *Pertussis* examination was 8.46, while the average for the RMSF examination was 8.29. The score range for the *Pertussis* exam was 5 to 10, whereas scores for the RMSF exam ranged from 2 to 10.

Regarding the opinion survey administered to the residents, a summary of the results is presented in Figure 1. All participants (100%) reported that the examination was well written and that the language used was coherent. Additionally, 34 residents (97.14%) stated that the language was clear, concise, and easy to understand. However, when asked if the language used was confusing 9 residents (25.71%) agreed, while 26 (74.29%) disagreed. In terms of the difficulty of the exam questions, 33 residents (94.28%) rated them as difficult. Finally, 32 residents (91.42%) felt that they had adequately prepared for both examinations.

## 4 Discussion

To our knowledge, this is one of the first documented studies involving medical residents in Mexico that describes the experience of faculty using ChatGPT as a tool to support teaching activities in the training of medical specialists. Previous studies have reported the use of LLMs for generating examination questions in specialties such as otolaryngology and emergency medicine (Lotto et al., 2024; Law et al., 2025), demonstrating the model’s strong ability to formulate multiple-choice questions. Moreover, a systematic review has evaluated the efficacy of LLMs in creating high-quality multiple-choice questions (Kıyak and Emekli, 2024).

Although no statistical analysis was performed in our study, the results provide preliminary evidence of the model’s ability to generate complex questions suitable for postgraduate-level health education. Notably, most residents considered the questions generated by ChatGPT to be difficult, indicating that they could represent a cognitive challenge comparable to questions traditionally developed by experienced medical professors. This underscores the importance of prompt quality (Kıyak, 2024), as the AI’s output quality strongly depends on the input provided. Nevertheless, the integration of AI tools like ChatGPT in hospital-based educational activities either for teaching or resident learning remains understudied, and it is still unclear whether statistically significant educational benefits exist compared to conventional teaching strategies.

Our findings align with prior reports of student acceptance and satisfaction regarding the quality of AI-generated questions (Rivera-Rosas et al., 2024). Similar studies have reported high levels of user satisfaction among medical students using AI for various academic tasks (Boris Miclin et al., 2024). Otherwise, medical professors’ opinions should also be evaluated in residence training contexts to overview their acceptance or rejection of its usefulness and their perceived knowledge about this tool and how to use them in their teaching activities in the hospital.

In this study, we highlighted some empirical uses of AI within hospital settings for specialist training, ranging from question generation for assessments to the integration of smart platforms such as Google Forms. These tools in addition to other AI models could offer two key advantages: (a) reducing the human workload of healthcare personnel that participates in residence training, and (b) becoming integral to the next phase of medical education through AI-assisted content creation supervised by professors.

While our work focuses specifically on ChatGPT’s utility in generating MCQ, further exploration of other applications is warranted. These may include drafting high-quality clinical notes, assisting with emergency department triage, creating presentations, enhancing scientific literature searches, or translating scientific articles applications that have been discussed in other studies (Hallquist et al., 2025). These use cases could indirectly reduce physician workload, improve learning outcomes, and enhance patient care. Otherwise, professors using LLM as ChatGPT should be aware that the knowledge (or “AI training), accuracy, context recognition and the ability of handling more complex prompts can vary depending on the LLMs version used for the MCQ creation, as their capabilities for answering or generating MQC can be vary (Mistry et al., 2024; Liu et al., 2025). This supports the needed supervision and domain that professors should have of AI tools for its adequate uses.

Despite the promising outlook, it is essential to remain aware of the limitations of AI, including ethical concerns such as plagiarism and authorship attribution in scientific writing, as well as philosophical questions regarding AI’s impact on critical thinking and its susceptibility to generating factual inaccuracies or “hallucinations” (Jeyaraman et al., 2023). Among the limitations of our study is its exploratory nature. We did not perform statistical validation of the AI-generated questions. Also, our results display promising utility of this AI tool in creating difficult MCQ, which also could diminish workload for teachers, but a quality comparison against human questions in medical residence scenarios should be assess in prospective studies to clarify its validated utility. Therefore, further research with greater scientific rigor are required to substantiate our findings.

## 5 Conclusion

Artificial intelligence and large language models like ChatGPT have become part of our new reality. Increasing evidence supports their potential benefits in the medical field, from education to clinical care. For this reason, their integration into healthcare processes should not be resisted but rather approached pragmatically as tools to be used thoughtfully. Developing AI literacy and competencies among medical students and faculty is essential and should progressively be incorporated into medical curricula to better prepare professionals for AI-assisted medical education and clinical practice.

## Data Availability

The original contributions presented in the study are included in the article/Supplementary material, further inquiries can be directed to the corresponding author.
