# Supplementary material for: Using ChatGPT as an assessment tool for medical residents in Mexico: a descriptive experience
Source: Front Artif Intell. 2025 Sep 15;8:1662203. doi: 10.3389/frai.2025.1662203 (PMC12477133; doi:10.3389/frai.2025.1662203)
Supplement: Supplementary file 1 [file Table_1.DOCX]

Supplementary Material

# Supplementary Table 1. Questions generated by ChatGPT

| **Topics** | **Questions** | **Answers options creates by Chatgpt** |
| --- | --- | --- |
| Pertussis | 1. What is the main causative agent of pertussis (whooping cough)? | A) Haemophilus influenzae **B) Bordetella pertussis**  C) Streptococcus pneumoniae D) Mycoplasma pneumoniae |
|  | 2. Which other Bordetella species can cause a pertussis-like syndrome? | **A) Bordetella parapertussis**  B) Bordetella bronchiseptica C) Bordetella holmesii D) All of the above |
|  | 3. What is the main mechanism by which Bordetella pertussis causes disease? | A) Direct invasion of the lung parenchyma **B) Toxin production that damages the respiratory epithelium**  C) Formation of granulomas in lung tissue D) Immune suppression leading to secondary infections |
|  | 4. What is the hallmark symptom of the paroxysmal stage of pertussis? | A) Persistent dry cough **B) Cough with a characteristic “whoop”**  C) Productive cough with purulent sputum D) Barking cough similar to croup |
|  | 5. Which of the following is a key feature of the catarrhal stage of pertussis? | A) Sudden high fever and chills **B) Rhinorrhea, mild cough, and conjunctival injection**  C) Severe pneumonia with dyspnea D) Petechial rash on the trunk |
|  | 6. What complication is most commonly associated with pertussis in infants? | A) Pulmonary embolism **B) Apnea and respiratory failure** C) Pulmonary fibrosis D) Chronic bronchitis |
|  | *7. Which age group has the highest risk of complications from pertussis? | A) School-age children (6-12 years) B) Adolescents (12-18 years) C) Adults over 50 years **D) Infants under 6 months** |
|  | 8. What is the gold standard for diagnosing pertussis? | A) Chest X-ray **B) Culture of nasopharyngeal secretions**  C) Complete blood count with differential D) Serology for pertussis toxin |
|  | 9. Which laboratory finding is commonly associated with pertussis in infants and young children? | A) Neutropenia **B) Lymphocytosis** C) Eosinophilia D) Monocytosis |
|  | *10. What is a characteristic finding on a chest X-ray of a patient with pertussis? | A) Patchy alveolar infiltrates **B) Hyperinflation with peribronchial thickening**  C) Pleural effusion D) Cavitary lesions |
|  | 11. What is the first-line antibiotic for treating pertussis? | **A) Azithromycin**  B) Amoxicillin C) Ceftriaxone D) Doxycycline |
|  | 12. What is the most effective method of preventing pertussis? | A) Early antibiotic prophylaxis **B) Vaccination with the DTaP/Tdap vaccine**  C) Isolation of symptomatic individuals D) Use of face masks in public places |
|  | *13. At what ages is the DTaP vaccine routinely administered in children? | **A) 2, 4, 6 months; 15-18 months; 4-6 years**  B) At birth and then every 6 months until age 5 C) 6 months, 12 months, and 5 years D) Only after the age of 2 |
|  | *14. Why are adolescents and adults recommended to receive the Tdap booster? | A) To prevent tetanus infections **B) To boost immunity and reduce transmission to infants**  C) Because childhood vaccination is ineffective D) To eliminate bacterial carriage in the nasopharynx |
|  | *15. In which clinical situation should post-exposure antibiotic prophylaxis be given? | A) Only if symptoms develop after exposure **B) In all household contacts of a confirmed pertussis case**  C) Only in immunocompromised individuals D) Only in infants under 1 year old |
| RMSF | 1. What is the etiologic agent of Rocky Mountain spotted fever? | A) Rickettsia prowazekii **B) Rickettsia rickettsii**  C) Coxiella burnetii D) Bartonella henselae |
|  | 2. What is the primary vector of Rickettsia rickettsii in the Americas? | A) Cat flea (Ctenocephalides felis) **B) Dermacentor tick**  C) Aedes aegypti mosquito D) Body louse (Pediculus humanus corporis) |
|  | 3. What distinguishes bacteria of the Rickettsia genus? | A) They are Gram-positive cocci **B) They are obligate intracellular bacilli**  C) They produce spores in adverse conditions D) They grow in common culture media |
|  | 4. What is the most common initial symptom of Rickettsia rickettsii infection? | A) Petechial rash **B) Severe headache and fever**  C) Hepatosplenomegaly D) Dry cough and dyspnea |
|  | *5. What is the characteristic pattern of the rash in Rocky Mountain spotted fever? | A) Starts on the trunk and spreads to the extremities **B) First appears on the palms and soles**  C) Is maculopapular without progression D) Is limited to the face and neck |
|  | 6. What is a severe complication of untreated Rocky Mountain spotted fever? | A) Myocarditis and arrhythmias **B) Multiorgan failure and disseminated intravascular coagulation** C) Osteomyelitis due to hematogenous spread D) Hepatic abscesses |
|  | *7. Which of the following clinical findings suggests a more severe rickettsial infection? | A) Fever below 38°C (100.4°F) B) Normal blood pressure **C) Thrombocytopenia and neurological involvement**  D) Localized rash without systemic symptoms |
|  | 8. What is the most commonly used laboratory test to confirm the diagnosis of Rocky Mountain spotted fever? | A) Culture in cellular media **B) Indirect immunofluorescence assay (IFA)**  C) Blood culture on chocolate agar D) Mantoux test |
|  | 9. What is a common finding in the complete blood count of patients with Rickettsia rickettsii infection? | A) Leukocytosis with neutrophilia **B) Leukopenia with thrombocytopenia**  C) Marked eosinophilia D) Absolute lymphocytosis |
|  | *10. What liver function abnormalities can be found in severe rickettsial infections? | **A) Elevated transaminases**  B) Hyperalbuminemia C) Decreased bilirubin levels D) No significant alterations |
|  | 11. What is the drug of choice for treating Rocky Mountain spotted fever? | A) Amoxicillin **B) Doxycycline**  C) Ceftriaxone D) Azithromycin |
|  | *12. In which age group is doxycycline usually contraindicated, but permitted in severe rickettsial infections? | A) Infants under 6 months **B) Children under 8 years**  C) Elderly adults D) Pregnant women |
|  | *13. Which of the following is a key measure in preventing Rickettsia rickettsii infections? | **A) Use of repellents and tick control**  B) Administration of a Rickettsia vaccine C) Use of specific immunoglobulin D) Isolation of infected patients |
|  | 14. In a patient suspected of having Rocky Mountain spotted fever, when should antibiotic treatment be initiated? | A) Only after confirmation by serology B) After 5 days of symptoms **C) Immediately, without waiting for confirmation**  D) If the rash is purpuric |
|  | 15. What is the primary mode of transmission of Rocky Mountain spotted fever? | A) Rodent bites B) Contact with secretions from infected patients **C) Bites from infected ticks**  D) Drinking contaminated water |
| RMSF: Rocky Mountain Spotted Fever; *Questions that were eliminated from the exams; In bold the correct answer of each question. | | |
